# Hospital Onset Vancomycin-Resistant Enterococci Infections in a Northern California Tertiary-Care Center From 2019 to 2020

**DOI:** 10.1017/ash.2021.140

**Published:** 2021-07-29

**Authors:** Stephanie Rasmussen, Sarah Waldman

## Abstract

**Background:** Understanding the epidemiology and risk factors for nosocomial infections with vancomycin-resistant enterococci (VRE) is necessary for the prevention and control of VRE infections in the hospital setting. We sought to determine the incidence of nosocomial infections of VRE and to ascertain predictors associated with nosocomial infection. **Methods:** In this retrospective cohort study, data were collected from patients with VRE infection from January 2019 to December 2020 at a tertiary-care center in northern California. VRE infections were designated as hospital onset (HO) if the specimen was collected >3 days after hospital admission or community onset (CO) if the specimen was collected ≤3 days after admission. Associations between HO infections with time, unit, and specimen were identified. **Results:** Over the 2-year period, 214 unique VRE infections were identified in hospitalized patients; 115 infections were CO and 99 were HO. HO-VRE were associated most frequently with stay in medical/telemetry units (68%), followed by oncology–transplant units (15%) and ICUs (12%). There were ~4.7 and ~3.6 HO-VRE infections per month in 2019 and 2020, respectively. No differences were identified between HO-VRE infections in 2 medical units regarding glycopeptide and cephalosporin use in those units. The sources of VRE infections were urinary 45%, bloodstream 15%, stool 10%, and other 30%. Of the 45 infections in urine, 51% were identified from catheters (Foley and straight) and 27% were identified from clean-catch urine. Interestingly, 71% of patients with VRE identified from urine did not report urinary tract infection (UTI) symptoms at the time of collection. Urine was most often collected for urinalysis and culture from patients with nonspecific symptoms such as fever, leukocytosis, hypotension, tachycardia, or altered mental status. All urine collected from patients who reported UTI symptoms grew >100,000 CFU/mL in culture, while only 75% of cultures from patients without symptoms grew >100,000 CFU/mL. The most common antibiogram was resistance to ampicillin, cefazolin, levofloxacin, minocycline, penicillin, tetracycline, and/or vancomycin (42% of cases) and susceptibility to both daptomycin and linezolid (60% of cases). **Conclusions:** HO-VRE infections were frequently identified with urinary sources and were often associated with catheter use. However, the frequent lack of concurrent UTI symptoms suggests VRE colonization rather than infection in many cases. Understanding the epidemiology and risk factors for HO-VRE infections is essential for developing infection prevention protocols to reduce the incidence of those infections.

**Funding:** No

**Disclosures:** None

